# Retinal Remodeling and Metabolic Alterations in Human AMD

**DOI:** 10.3389/fncel.2016.00103

**Published:** 2016-04-28

**Authors:** Bryan W. Jones, Rebecca L. Pfeiffer, William D. Ferrell, Carl B. Watt, James Tucker, Robert E. Marc

**Affiliations:** ^1^Department of Ophthalmology, Moran Eye Center, University of UtahSalt Lake City, UT, USA; ^2^Interdepartmental Program in Neuroscience, University of UtahSalt Lake City, UT, USA; ^3^Department of Ophthalmology, University of California, DavisDavis, CA, USA

**Keywords:** age-related macular degeneration (AMD), retinal pigment epithelium (RPE), computational molecular phenotyping (CMP), retina, photoreceptor, Müller cell, retinal remodeling, neural remodeling

## Abstract

Age-related macular degeneration (AMD) is a progressive retinal degeneration resulting in central visual field loss, ultimately causing debilitating blindness. AMD affects 18% of Americans from 65 to 74, 30% older than 74 years of age and is the leading cause of severe vision loss and blindness in Western populations. While many genetic and environmental risk factors are known for AMD, we currently know less about the mechanisms mediating disease progression. The pathways and mechanisms through which genetic and non-genetic risk factors modulate development of AMD pathogenesis remain largely unexplored. Moreover, current treatment for AMD is palliative and limited to wet/exudative forms. Retina is a complex, heterocellular tissue and most retinal cell classes are impacted or altered in AMD. Defining disease and stage-specific cytoarchitectural and metabolic responses in AMD is critical for highlighting targets for intervention. The goal of this article is to illustrate cell types impacted in AMD and demonstrate the implications of those changes, likely beginning in the retinal pigment epithelium (RPE), for remodeling of the the neural retina. Tracking heterocellular responses in disease progression is best achieved with computational molecular phenotyping (CMP), a tool that enables acquisition of a small molecule fingerprint for every cell in the retina. CMP uncovered critical cellular and molecular pathologies (remodeling and reprogramming) in progressive retinal degenerations such as retinitis pigmentosa (RP). We now applied these approaches to normal human and AMD tissues mapping progression of cellular and molecular changes in AMD retinas, including late-stage forms of the disease.

## Introduction

Given that age-related macular degeneration (AMD) is effectively a deafferentation of the neural retina caused by the death of photoreceptors, our goal with this study was to explore whether or not AMD retinas exhibited the same retinal plasticity and remodeling observed in retinitis pigmentosa (RP; Li et al., 1995; de Raad et al., 1996; Fletcher and Kalloniatis, 1996; Fariss et al., 2000; Machida et al., 2000; Strettoi and Pignatelli, 2000; Strettoi et al., 2002, 2003; Jones et al., 2003, 2005, 2006, 2011, 2012; Marc and Jones, 2003; Marc et al., 2003, 2005, 2007, 2008; Cuenca et al., 2004; Jones and Marc, 2005; Pu et al., 2006; Aleman et al., 2007). AMD, like RP is a collection of defects. In AMD, these defects arise from from identified defects in CFH (Boon et al., 2009), ARMS2 (Fritsche et al., 2008; Friedrich et al., 2011), HTRA1 (Dewan et al., 2006), oxidative stress (Kunchithapautham et al., 2014) and inflammation (Ozaki et al., 2014) that ultimately result in pathologies manifesting from the molecular levels to tissue levels. Ultimately however, in both dry and wet forms of AMD, photoreceptors die which we hypothesized initiates the same cascade of neural cell death and plasticity observed in other retinal degenerative diseases such as RP.

For this study, we applied a set of technologies that reveal a metabolic “fingerprint” for cells while preserving all anatomical relationships. These approaches, computational molecular phenotyping (CMP) parse tissues into metabolic space, revealing structure in addition to metabolism. This study revealed fundamental and previously unknown findings including alterations in metabolic stability of retinal pigment epithelium (RPE) cells, particularly those above regions of pathology. We show evidence of photoreceptor cell stress that occurs prior to cell death and indications that cone opsin processing may be differentially compromised vs. rod opsin processing, specifically in AMD as compared with other retinal degenerative diseases. Being able to visualize metabolism is a powerful feature of this study as we’ve documented metabolic alterations in Müller cells which is a novel finding for AMD, even though it has been described for other retinal degenerative diseases. However, the most significant finding of this study is the extensive description of negative retinal plasticity, termed retinal remodeling that involves inner retinal neurons projecting to aberrant locations. This remodeling occurs underneath obvious regions of pathology like underneath drusen, but also in regions where cone and rod photoreceptors are still present suggesting implications for altered retinal processing prior to photoreceptor cell death.

## Materials and Methods

### Human Tissue

Human AMD tissue was obtained within 6 h post mortem from The Foundation Fighting Blindness Retina Donor Program, at the University of Utah Lions Eye Bank. Institutional approval for use of human eyes was obtained from the University of Utah and followed the tenets of the Declaration of Helsinki. All retinal tissues and data were de-identified in accordance with HIPPA Privacy Rules. Retinas from patients with a diagnosis of AMD were identified from both medical records and by post-mortem examination of globes, retinas were photographed and gross pathological features were documented. Five millimeter wide strips were dissected out starting at the optic nerve head and traversing horizontally through the macula and out to the temporal ora serrata. Portions of the strips collected for histological purposes exhibiting regions of pathology (drusen, hypopigmentation, neovascular growth) were trephine punched, prepared and processed for both light and electron microscopic analyses. For CMP, eyes are immersion-fixed overnight in buffered 2.5% glutaraldehyde/1% formaldehyde and resin embedded and serially sectioned at 70–250 nm (Marc et al., 1995). Parafoveal and mid-peripheral retina was utilized for the CMP in this study because many small molecule metabolic signals in the post-mortem fovea degrade more rapidly than peripheral retina for unknown reasons that may relate to increased metabolic demands (Napper and Kalloniatis, 1999). Retinas were obtained from three normal, control human subjects and eight AMD subjects. Six of the AMD subjects had diagnoses of dry AMD and two had diagnoses of wet AMD, one early in the course of the disease and one late in the progression of the disease.

### Non-Human Primate Tissue

Eyes from adult male and female olive baboons (*Papio anubis*) were obtained during necropsy from the Southwest Foundation for Biomedical Research (San Antonio, TX, USA). Anesthesia and euthanasia conform to institutional animal care and use authorizations and the ARVO Statement for the Use of Animals in Ophthalmic and Visual Research.

### OCT

Enucleated globes with the anterior segment removed were submerged post-fixation in a large scintillation chamber filled with normal saline for high resolution mapping and correlation with histological/CMP analysis. High resolution scans were performed with a Heidelberg Spectralis, OCT.

### Immunocytochemistry

Retinal neurons were classified by CMP (Marc and Jones, 2002). To generate signals for CMP, tissues were probed with IgGs selective for individual small or macro molecules [aspartate (D), arginine (R), glutamate (E), glycine (G), glutathione (J), glutamine (Q), taurine (τ), γ-aminobutyric acid (GABA; γ), CRALBP, rod opsin (1D4), cone opsin (rg-opsin) or glutamine synthetase (GS; Table 1], visualized with secondary antibodies conjugated to 1.4 nm gold, followed by silver intensification (Marc et al., 1995; see Table 1). Primary antibody incubations was performed overnight at room temperature and visualized with goat anti-rabbit secondary IgG coated with 1.4 nm gold (Nanoprobes Nanogold^®^ -anti Rabbit IgG) and silver intensified for CMP (Marc et al., 1995). All probed signals derive from 200 nm thick sections and all epitope binding of antibodies occurs in the first 5 nm from the surface of the section, making all signals quantitative (Kalloniatis and Fletcher, 1993; Marc et al., 1995).

**Table 1:** **List of antibodies**.

Reagent	SKU	RRID	Source	Dilution
anti-L-aspartate IgG	D100R	AB_2341093	Signature immunologics	1:100
anti-L-glutamate IgG	E100R	AB_2532055	Signature immunologics	1:100
anti-glycine IgG	G100R	AB_2532057	Signature immunologics	1:100
anti-glutathione IgG	J100R	AB_2532058	Signature immunologics	1:100
anti-L-glutamine IgG	Q100R	AB_2532059	Signature immunologics	1:100
anti-taurine IgG	TT100R	AB_2532060	Signature immunologics	1:100
anti-GABA IgG	YY100R	AB_2532061	Signature immunologics	1:100
anti-GS IgG	610517	AB_397879	BD Biosciences	1:50
anti-CRALBP IgG	NA	AB_2314227	Gift of Dr. Jack Saari	1:400
anti-rod opsin 1D4 IgG	NA	AB_2315015	Gift of Dr. Robert Molday	1:4000
anti-cone opsin IgG	AB5405	AB_177456	EMD Millipore	1:1000

### Computational Molecular Phenotyping (CMP)

Images were captured as 8-bit high-resolution (243 nm/pixel) images (Marc and Jones, 2002), mosaicked, and registered with ir-tweak https://www.sci.utah.edu/download/ncrtoolset.html into large image databases. Cell classification was performed on N-dimensional (N-space) monochrome images via k-means or Iterative self-organizing data analysis technique (ISODATA) clustering (Marc et al., 1995; Marc and Cameron, 2001; Marc and Jones, 2002) using PCI Geomatica (PCI Geomatics, Inc.) for pixel based clustering and mask generation into theme maps. Detailed theme map generation first involves production of raw classification theme maps (Marc et al., 1995; Kalloniatis et al., 1996), which is the mathematical division of regions into statistically separable classes based on multiple channel inputs. Adobe Photoshop (Adobe, San Jose, CA, USA) was used for final image generation. For display only, raw data channels are linearly contrast-stretched over a 30–220 pixel value range and sharpened with unsharp masking. Molecular signals were visualized as selected rgb maps encoding three molecular signals as red, green, and blue, respectively, e.g., γ.G.E → rgb which assigns GABA, glycine and L-glutamate to red, green, and blue color channels, respectively. Monochrome images are density mapped and rgb images intensity mapped (Marc et al., 1995). Both high and low magnification electron microscopy (EM) images montages are captured digitally as 12-bit monochrome channels and assembled into large mosaics (Anderson et al., 2009).

### Electron Microscopy (EM)

Tissues were postfixed in 1% buffered osmium tetroxide, followed by resin embedding. Large scale transmission electron microscopy (TEM) was then performed, creating EM mosaics as previously described (Anderson et al., 2009) on 90-nm lead-stained sections on single-slot grids.

## Results

We display aged and AMD tissues with both individual gray scale and rgb mapping reflecting small molecule concentrations. Mapping of signals as γ.G.E → rgb triplets allows visualization of large swaths of tissue for rapid review, such as GABA (γ) and glycine (G) amacrine cells, photoreceptors, bipolar cells, and ganglion cells, whereas the mapping of τ.Q.E → rgb sets apart Müller cells, the retinal pigmented epithelium, and other elements from all other cells. Human retina shows essentially the same composite patterns of small-molecule signatures as those in the primate, cat, rabbit and rat retinas (Fletcher and Kalloniatis, 1996; Kalloniatis et al., 1996; Marc et al., 1998b; Marc, 1999c). Classifying all the data with k-means or ISODATA clustering produces raw theme maps, which are then converted to refined theme maps superimposed on a single reference channel. Theme maps and rgb displays simplify tracking of 10 major cell phenotypes; photoreceptors, horizontal cells, ON-center cone bipolar cells identified as glycine (G) bipolar cells (Kalloniatis et al., 1996), all remaining bipolar cells as a mixed group of rod and OFF-center cone bipolar cells, glycine (G) amacrine cells, GABA (γ) amacrine cells, ganglion cells, Müller cells, vascular elements, and the retinal pigmented epithelium. These 10 phenotypes account for effectively all retinal space.

Human tissues were harvested within 3–5 h post mortem for analysis. Small molecules are robust signals that can be used for discrimination of individual cell classes (Marc and Liu, 1985; Marc et al., 1990, 1995; Kalloniatis et al., 1996; Marc, 1999a,b; Marc and Cameron, 2001; Marc and Jones, 2002) but some signals are impacted by ischemia. Redistributions in small molecules due to neuronal reverse transport and glial accumulation are detectable within minutes of ischemia in retina, causing clear banding in the IPL and increases of GABA in the Müller cells. GABA signals in the peripheral retina are better preserved than in central retina. With increasing time, barring re-perfusion, GABA builds up in Müller cells, while other small molecule signals remain intact. While it is difficult to obtain human tissues within a window where GABA signals do not show alterations, this does not interfere with cellular class identification.

**Figure 1:**
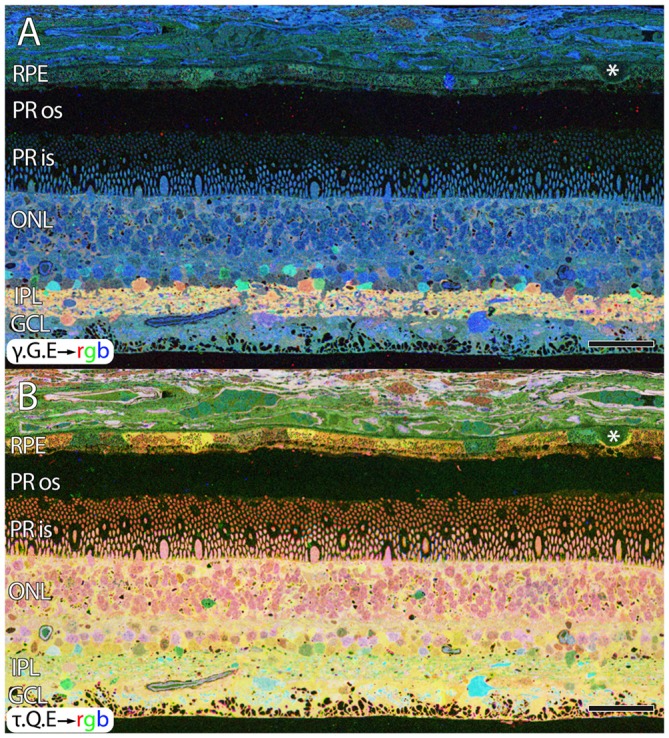
**Normal aged, peripheral human retina. (A)** γ.G.E → rgb mapping of human peripheral retina from a 78 year old male with no diagnosis of AMD. Normal topology and stratification of the retina is observed, but the earliest indications of potential pathology are present in the bricking of the retinal pigment epithelium (RPE), a common finding in aged, human retina. Also, note the existence of a small druse, labeled with asterisk. **(B)** τ.Q.E → rgb mapping revealing Müller glial populations in yellow. Scale bar = 40 μM.

Aside from these alterations in GABA, aged human retinas show normal retinal stratification and organization. However, many aged normal retinas (e.g., Figure 1), with no previous diagnosis of AMD exhibit pathologies in the RPE that are suggestive of changes due to aging or even incipient AMD (pre-diagnosis) with the identification of occasional small, hard druse, (asterisk in Figure 1) though isolated druse are not thought to be associated with age related maculopathy (Sarks et al., 1999).

### RPE

Metabolic signatures in individual RPE cells of normal tissues are indistinguishable from each other. This has been true in our analysis of over 1000 retinal samples from at least 70 vertebrate species, but patches of RPE with variable signals are common in AMD retinas. We term this pattern “bricking, ” similar to a running bond course with two colors of bricks. In normal RPE cells, levels of amino acids are consistent and uniform. In AMD samples, levels of taurine, glutamate, and glutathione in the RPE demonstrate wide variability across immediate neighbors, notably in the macula. However, occasional tiling of the RPE is not uncommon in aged human samples (Figure 1), and could be indicative of normal aging. But it also may be an early manifestation of AMD or an explicit risk phenotype for AMD. Regardless, extensive bricking of RPE cells correlates with the presence of other pathologies associated with AMD. More advanced cases of AMD show dramatic alterations, particularly where RPE cells are near drusen deposits. Critically, the neural retina directly under deposits is remodeled and a cascade of pathology spanning photoreceptors, Müller cells and eventually, neurons in the retina are all metabolically and/or structurally compromised.

**Figure 2:**
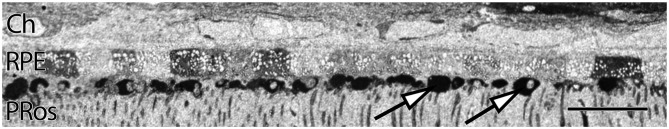
**Human parafoveal retina from a 76 year old patient with advanced AMD showing taurine labeling in the RPE internal to the vascular choroid (Ch).** Normal levels of taurine in the RPE are typically high and uniform. As RPE cells experience cell stress, they adopt a “bricked” or non-uniform appearance suggesting that they are becoming uncoupled. While this pathology is present to a limited degree in ostensibly normal aged human retina, it becomes much more dramatic in regions of obvious AMD related pathology. The white spots in the RPE likely indicate regions of lipid accumulation and could represent lipofuscin/melanolipofuscin granules. Also of note, are the accumulations of intensely taurine positive deposits underneath the RPE and above the photoreceptor outer segments (arrows). These deposits are not apical processes of the RPE as they have much higher concentrations of taurine than do the RPE cells. Scale bar = 40 μM.

RPE expresses uniform, moderate levels of glutathione, a redox control molecule. In most species the concentration ranges from 1 to 5 mM, with all RPE cells in a sample showing the same level. Glutathione levels also tend to decrease in concentration with eccentricity in normal retinas, but exhibits marked increases in the central retina of patients with diagnoses of AMD. Additionally, RPE glutathione levels are heterogeneous in AMD retinas, as are other small molecules expressed in the RPE, including glutamate and taurine. (Figure 2). Individual RPE cells from AMD patients often demonstrate paradoxically dramatically lower or higher levels of both glutathione and taurine in unpredictable patterns, in addition to a dramatic loss of other central metabolic carbon skeleton amino acids such as core metabolites such as glutamate, which may be a signature of incipient cell death.

**Figure 3:**
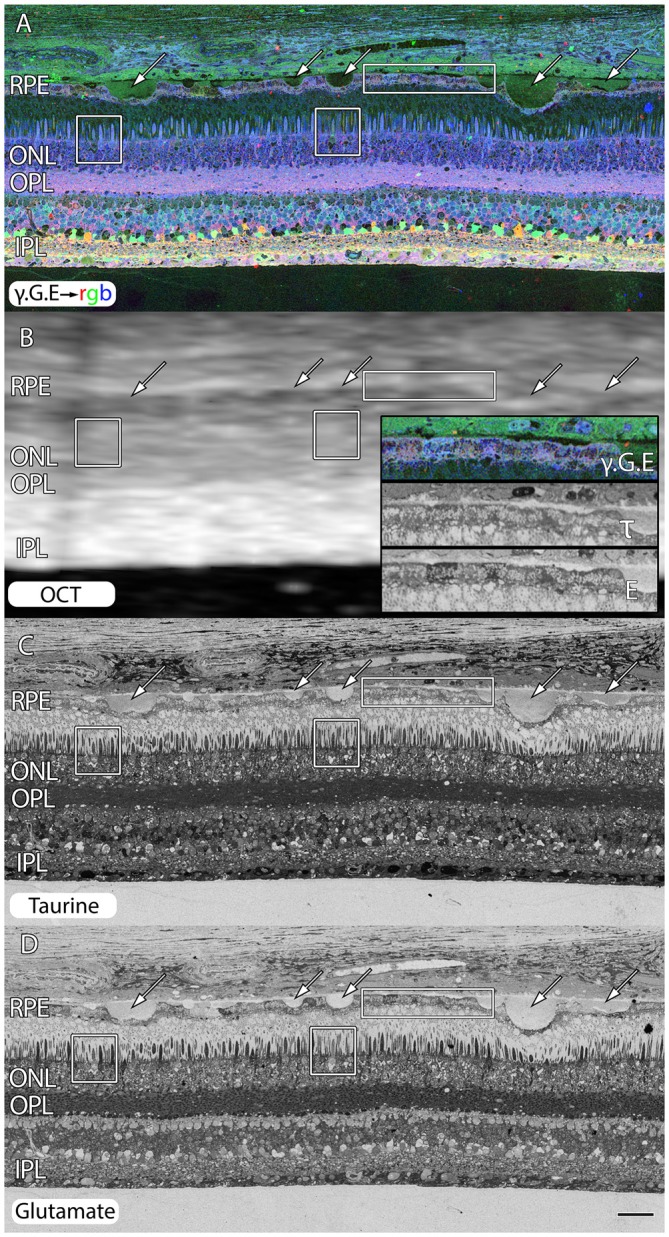
**Peripheral retina from a 71 year old AMD patient demonstrating small and mid-size drusen (arrows), and RPE bricking particularly underneath large druse. (A)** γ.G.E → rgb. **(B)** Post-mortem *ex-vivo* OCT data demonstrating correlates with histology in **(A,C,D)** with inset demonstrating higher magnification views of RPE histology shown in rectangle. **(C)** Taurine labeling demonstrating RPE bricking. **(D)** Glutamate labeling demonstrating RPE bricking and altered cone photoreceptors (boxes) revealing cones with low glutamate concentrations. Scale bar = 80 μM.

Even under small drusen, RPE cells are “bricked”. Figure 3 shows an area of retina that is largely preserved from a 71 year old patient with advanced AMD. However, all of the RPE cells around these drusen (arrows) are clearly compromised with extensively altered metabolism shown in the rectangles and inset of Figure 3B. We also include post-mortem *ex-vivo* OCT data, co-registered to the histology in Figure 3B to demonstrate features such as the small 20–60 μM drusen seen in histology are difficult to assess in post mortem OCT and that the other changes being shown indicative of pathologies in retinas might be invisible to current commonly available clinical assessments.

**Figure 4:**
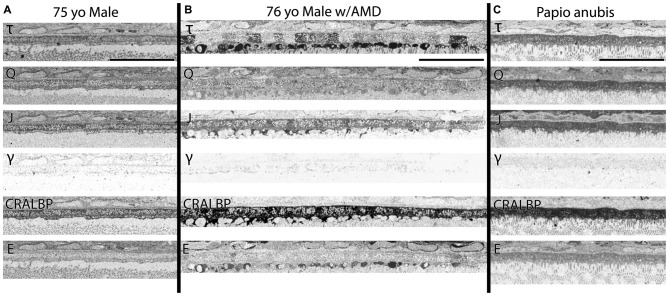
**(A)** RPE from a 75 year old male with no history of AMD. **(B)** A 76 year old male with AMD and **(C)** mature male Papio anubis. The RPE is shown in each column labeled for taurine (τ), glutamine (Q), glutathione (J), GABA (γ), CRALBP and glutamate (E). This image most notably demonstrates the variability in taurine content of the RPE cells compared with normal human and non-human primate but also demonstrates the lack of staining in GABA of the RPE indicating that any pigment granules in the RPE do not alter or influence small molecule epitope detection. Also, note that CRALBP is upregulated over the normal human in AMD and that a subset of the taurine deposits differentially contains glutamate as well. Scale bar = 80 μM.

**Figure 5:**
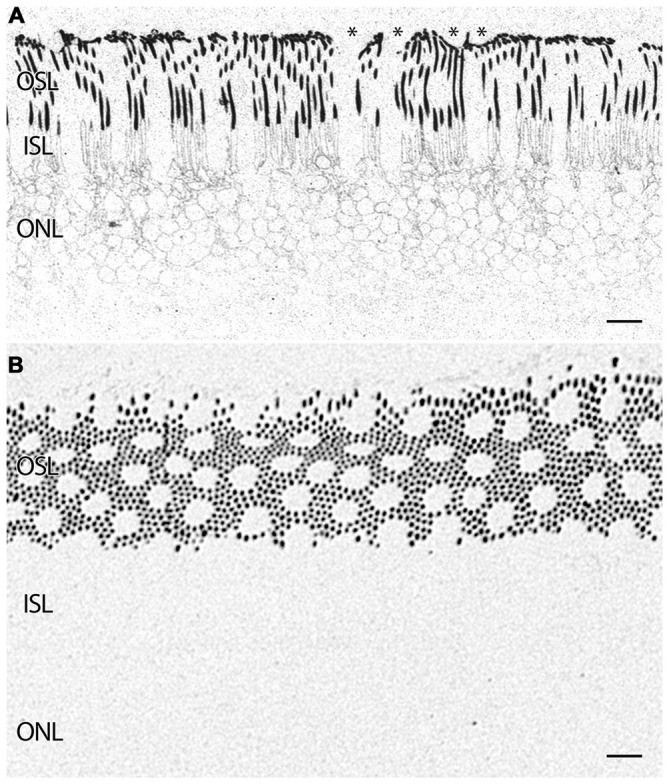
**(A)** Parafoveal retina from a 71 year old male patient, 2 h 41 min post mortem with a diagnosis of AMD labeled for rhodopsin demonstrating extensive rod opsin delocalization in the rod photoreceptors down around the inner segments and cell bodies. Asterisks denote small possible subretinal drusenoid desposits (SDD) or aberrant apical processes of RPE deforming the tips of the outer segments of photoreceptors. Notably, no rhodopsin buildup is occurring underneath the RPE as occurs in many retinitis pigmentosa (RP) diseases. **(B)** Demonstrating a slightly more oblique section of rhodopsin labeling in a normal parafoveal non-human primate retina revealing no rhodopsin delocalization. Scale bar = 8 μM.

The variability of labeling in RPE cells is not due to any alterations in pigment composition of the RPE across AMD samples, nor between regions of retina within samples seen in Figure 4. Regional variation in pigment has never been described on an RPE cell to cell basis and could not explain the variations in signatures observed in AMD samples, where variations in labeling in small molecule signals of glutamate, glutamine, (Figures 1, 3, 5) and taurine (Figures 2, 4, 5, 8, 9) within the RPE in unpredictable patterns is commonly seen. This is the case particularly underneath photoreceptors that appear with altered metabolism or other pathological findings in and around the photoreceptors such as the taurine positive deposits described in Figures 2, 4.

### Photoreceptors

**Figure 6:**
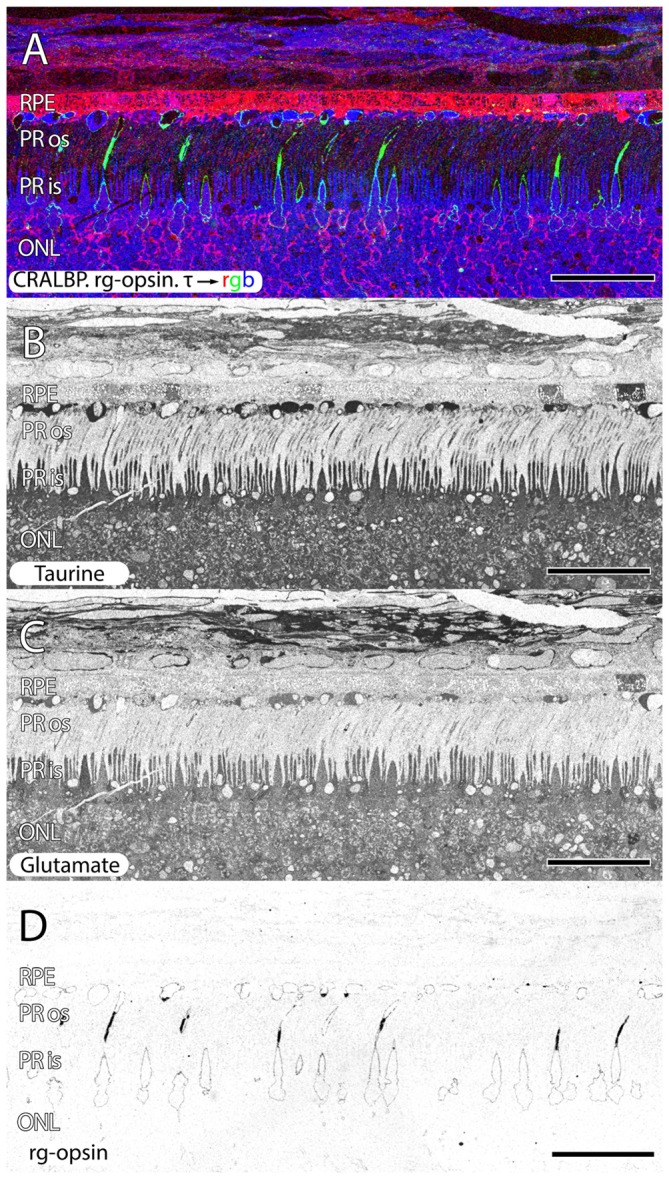
**Parafoveal retina from a 76 year old patient with AMD demonstrating CRALBP.rg-opsin.τ → rgb in (A), taurine labeling in (B), glutamate labeling in (C) and cone opsin in (D).** This image composite shows RPE bricking as well as cone opsin bounded taurine and glutamate rich deposits underneath the RPE. Cone opsin delocalization is also shown in **(A,D)**. Scale bar = 80 μM.

In addition to indications of cell stress in the RPE, retinas from patients with AMD demonstrate photoreceptor abnormalities. In some regions, rod photoreceptors demonstrate rhodopsin delocalization (Milam et al., 2002) underneath small ~10 μm diameter deformations that may represent subretinal drusenoid deposits (SDD) or aberrant apical processes of RPE cells that displace the outer segments of the rod photoreceptors (Figure 5A). Cone opsin, is clearly delocalized around the inner segments and cell bodies in regions where the RPE is bricked and builds up large deposits underneath those RPE cells filled with taurine and glutamate and ringed by cone opsins suggestive of RPE defects in both retinoid processing (Baehr et al., 2003) and perhaps cone-selective phagocytosis. Cone opsins also undergo cone opsin delocalization to a greater degree in the macula than in the mid periphery and exhibit altered glutamate signatures underneath druse suggesting challenged metabolism or cell stress, indicating that retinoid processing is compromised (Figures 3, 6). Figure 3D demonstrates cone photoreceptors with altered glutamate metabolism. These cone photoreceptors have far lower glutamate concentrations in them than the surrounding cone photoreceptors. At late stages of AMD in both dry and wet forms, rhodopsin is still present, yet cone opsins completely disappear. Since each cone photoreceptor is ensheathed by a single RPE cell (Gao and Hollyfield, 1992), defects in the RPE may be rapidly expressed in cones.

Another common, but structurally unusual finding is a buildup of taurine underneath the RPE in (Figures 2, 6) enveloped by a thin ring of membrane containing cone opsin. These deposits are a mix of taurine at very high concentrations, glutamate, aspartate, glutamine at lower concentrations and also appear to have lipid droplets associated with them. This may represent specific failure to cycle cone outer segments.

**Figure 7:**
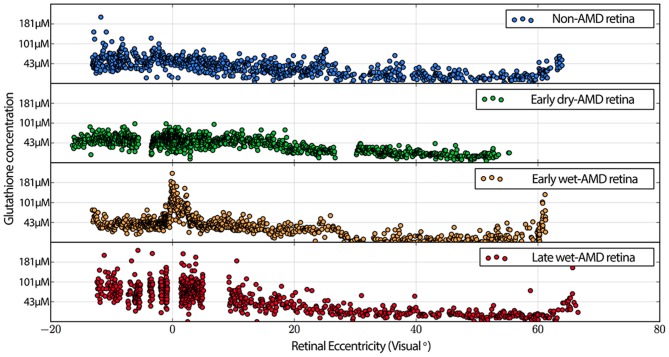
**Graphs demonstrating glutathione concentration in the inner segments of cone photoreceptors over retinal eccentricity in four example patients, normal, early-AMD, early wet-AMD, with neovascularization present, but prior to large scale bleeds in the retina and late wet-AMD with prevalent evidence of resolved retinal bleeding.** Normal glutathione concentration is very low, but becomes dramatically variable in central retina of wet-AMD. Each plot represents data from a single individual (*n* = 1) of normal, early, dry-AMD, early wet-AMD and late we-AMD.

Also commonly observed are isolated cone photoreceptors with dramatically lower levels of both taurine in RPE above cone photoreceptors that exhibit low levels glutamate suggesting the possibility of a late phase cell death signature. Additionally, cone inner segment glutathione profiles in normal retina demonstrate very little heterogeneity with very low concentrations that decrease to effectively zero in the peripheral retina. However in wet-AMD retinas, glutathione increases more than two-fold in the fovea/macula over surrounding areas and demonstrates heterogeneous concentrations. The increases in concentrations return to more normal levels past 20 degrees eccentricity (Figure 7). Cone inner segment taurine profiles show very high concentrations of taurine in them with little variation across all retinal eccentricities regardless of AMD status (data not shown).

### Müller Cells

**Figure 8:**
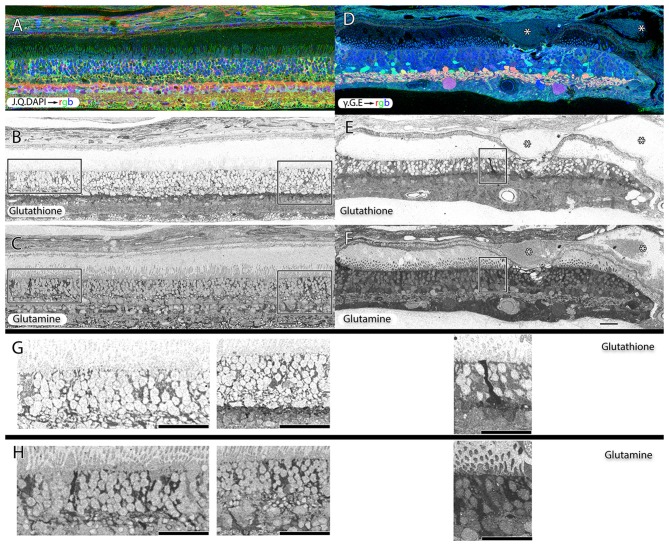
**Two retinas from 75 year old patients with early dry-AMD in (A–C) with J.Q.DAPI → rgb in (A), glutathione labeling in (B), glutamine labeling in (C), and mid to late stage wet-AMD in (D–F) with *γ*.G.E → rgb shown in (D), glutathione in **(E)** and glutamine in (F).** Boxes (higher magnification in **G,H**) show Müller glia that has dramatically elevated glutathione and glutamine signals in isolated or groups of glia. In the case of wet-AMD **(D–F)**, the ONL is dramatically thinner than in the early dry-AMD retina **(A–C)** and appears much more distinct because of this loss of photoreceptor cell nucleii. **(G,H)** Show increased magnification views of the boxes in **(B,C,E,F)** for glutathione and glutamine channels respectively. Scale bar = 80 μM. Asterisks demonstrate drusen (*).

Müller cells are one of the first cell classes in the retina to show metabolic alterations in retinal degenerations (Jones et al., 2003, 2005, 2006, 2011, 2012; Marc and Jones, 2003; Marc et al., 2003, 1995; Jones and Marc, 2005). AMD retinas resemble RP retinas in this regard. Glutamine and glutathione variability can be observed in Müller cells of patients with early dry-AMD, with spikes of more than double normal levels. This is also true in wet-AMD (Figure 8).

**Figure 9:**
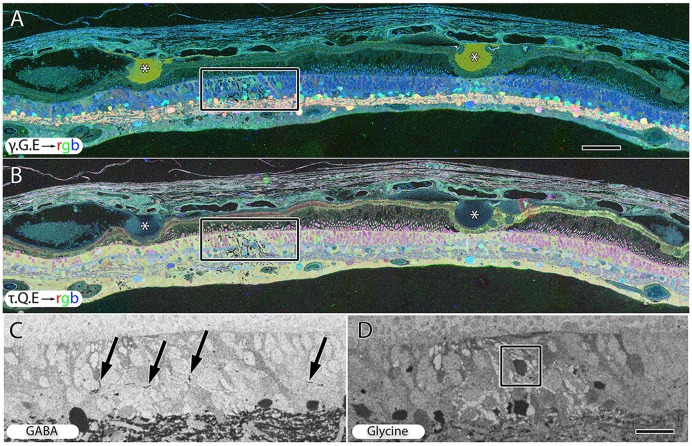
Peripheral retina from late stage dry AMD patient with drusen (*) demonstrating hypertrophy and metabolic alterations in Müller glial cells. (A) Shows γ.G.E → rgb and **(B)** shows τ.Q.E → rgb. The inset rectangles demonstrate magnified regions shown in **(C,D)** revealing GABAergic labeling of aberrant GABAergic processes (arrows) outside the normal lamination of the IPL in **(C)** and glycinergic labeling of a misplaced glycinergic amacrine cell in **(D)** demonstrating clear plasticity and remodeling in inhibitory neuronal classes. Note, in the post-mortem state, GABA and glycine increase in the Müller cells. Scale bar = 200 μM in **(A,B)**. Scale bar = 40 μM in **(C,D)**.

Hypertrophy of glial cells occurs as photoreceptor cell bodies in the outer nuclear layer (ONL) are lost (Figure 9). This may be a result of Müller cell distal processes remaining after the loss of photoreceptor cells in the ONL, or there may be actual hypertrophy as in RP and RP related retinal degenerations.

As AMD progresses in both wet and dry forms , there is a dramatic loss of GS labeling in Müller glia.

### Inner Retinal Neurons

Early to mid-stage AMD is commonly associated with numerous moderate to large drusen deposits in the sub-RPE space. GABA, glycine and glutamate signals display morphological changes in neurons directly under drusen (Figure 9A) while taurine, glutamine, glutamate signals (Figure 9B) shows that the Müller express metabolic variability in the same region. Ectopic neurites of GABAergic amacrine cells and translocation of glycinergic amacrine cells into the OPL and ONL and likely into the IPL, particularly in regions around moderate to large drusen formations, demonstrate that pathologic remodeling is both local and unexpectedly fast in AMD, occurring even while rod photoreceptors are present (Figures 9C,D). Neuronal remodeling has been extensively characterized in RP and RP models (Jones et al., 2003, 2005, 2006, 2011, 2012; Marc and Jones, 2003; Marc et al., 2003, 2008; Jones and Marc, 2005).

## Discussion

AMD affects an estimated 18% of Americans from 65 to 74 and 30% older than 74 while the risk accumulates with age. The single largest risk factor for AMD is age, but the pathways and mechanisms through which genetic and non-genetic risk factors modulate structural AMD pathogenesis remain largely unexplored. Moreover, current treatment for AMD is palliative and limited to exudative forms. While AMD represents one of the best characterized diseases from a genetic perspective, we currently know far less about the mechanisms mediating disease progression, and other novel methods for interrogating the anatomy and metabolism of retina are needed.

The goal of this work was to initiate an exploration of the metabolic status and track fates of RPE and retinal cells in retinas from patients with a diagnosis of AMD. There are multiple presentations of RPE cells based upon morphological analysis (Zanzottera et al., 2015) and the analysis presented here demonstrates a number of metabolic intermediate states that may reflect differential survival or stages of cell stress. Serial monochrome images of normal human retina probed for different small molecules display patterns of labeling similar to those of other mammals. However, no comprehensive analysis has yet been performed on aging retina to determine how these cell populations change in senescence or AMD. The question of aging is is beyond the scope of this manuscript, and we refer the reader to some notable studies on the histology (Gao and Hollyfield, 1992; Curcio et al., 1993; Samuel et al., 2011) and molecular biology (Barron et al., 2001; Louie et al., 2002) and genetics of ocular aging (Yoshida et al., 2002). Heterocellular metabolism appears to be stable over time, even in aged retina and across species (Marc et al., 1978, 1990, 1995, 1998b; Marc and Lam, 1981; Marc and Liu, 1985; Marc, 1986, 1989, 1992, 1999c, 2004, 2008; Kalloniatis et al., 1994, 1996; Marc and Cameron, 2001; Marc and Jones, 2002).

Peri-foveal tissue: we acknowledge that AMD is thought to be a macular disease, but are interested in the earliest stages of AMD and it is reasonable to look to the edges of presumptive healthy retina for the earliest manifestations of pathology. While rod loss is normal in aging (Parapuram et al., 2010), it is also accelerated in early AMD (Curcio, 2001) and there is no evidence that the disease process itself ceases at the boundary of the fovea or macula. Indeed, studies on protein expression changes in macula and periphery found that the majority of protein changes in AMD happened in the periphery (Ethen et al., 2006) and contrast sensitivity in the periphery is lower in AMD patients than in controls (Faria et al., 2015). Additionally, mitochondria in the peripheral RPE of AMD patients exhibits damage (Terluk et al., 2015) and investigators have explored clinically observable changes in the peripheral retinas of patients with AMD (Reznicek et al., 2012).

For these reasons, we believe the peripheral retina from AMD patients is a tremendous resource to understand disease progression.

### Visualization of Metabolism in AMD Retina with CMP

CMP allows us to visualize the metabolic state of the retina in health and disease, revealing pathologies that are undetected by other methodologies. For instance, the presence of variable concentrations of glutamate and taurine in RPE and cone photoreceptors suggests the possibility of a late phase cell death signature and the collapse of GS labeling in retinas of AMD patients is notable for its potential contribution to the metabolic alterations or chaos invoked, particularly in Müller cell populations.

Using CMP to examine AMD pathogenesis reveals cell metabolic state, and provides the ability to precisely define individual cell classes affected by disease processes while preserving the histologic and anatomic context. Thus, the use of CMP for assessing the metabolome of AMD provides important new biological information pertaining to metabolic diversity across cell classes, while demonstrating tight regulation of metabolic envelopes within cell classes (Marc et al., 1995; Kalloniatis et al., 1996; Marc and Cameron, 2001; Marc and Jones, 2002).

### Retinal Remodeling in AMD

**Figure 10:**
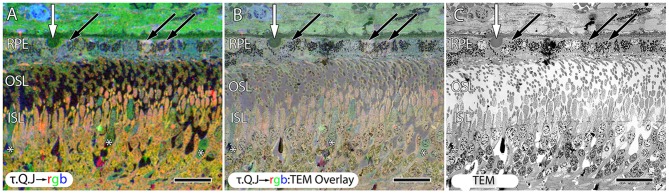
**Early stage dry AMD patient with very small drusen showing in τ.Q.J → rgb labeling, (A) and τ.Q.J → rgb overlay on top of transmission electron microscopy (TEM) imagery in (B) with pure TEM imagery in (C).** Black arrows demonstrate RPE cells with varying taurine, glutamine and glutathione concentrations. Asterisks demonstrate cone photoreceptors with altered metabolic signatures showing decreases in taurine, and elevations in glutamine. Vertical white arrows denote the presence of a small druse. Scale bar = 8 μM.

Early studies demonstrated photoreceptor loss in AMD (Curcio et al., 1996), and photoreceptor loss associated with drusen (Johnson et al., 2003). Additional studies have explored synaptic alterations and altered gene expression in photoreceptors (Johnson et al., 2005), and examined synaptic plasticity in AMD at all eccentricities (Sullivan et al., 2007). This article shows in AMD, that alterations of small molecule signatures of the RPE, cone photoreceptors and Müller cells indicate that early stress presages later photoreceptor loss and retinal remodeling (Figures 3, 6, 8–10), particularly underneath drusen (Figures 3, 9). This retinal remodeling is itself a likely cause of blindness, even before complete photoreceptor loss occurs and is a novel finding. Importantly, this remodeling occurs in the presence of rods. Retinal remodeling in RP occurs primarily in response to cone loss (Jones et al., 2003, 2005, 2006, 2011, 2012; Marc and Jones, 2003; Marc et al., 2003, 2005, 2007, 2008; Jones and Marc, 2005). Cones appear to stabilize the retina in RP while this work demonstrates that early cone stress appears to induce retinal neural network remodeling in the presence of cones, the mirror image of what is observed in early retinal reprogramming in RP.

Regardless of the precise triggering event(s) that provokes the subsequent RPE-retina pathology, it is clear that the major downstream consequences of these processes are the deposition and sequestration of cellular and acellular debris in the remnant sub-RPE space (drusen), photoreceptor cell degeneration and, eventually, concomitant loss of vision. Over the past decade, proteomic, molecular, genomic and mass spectroscopy-based metabolomic assays have revealed tremendous insight into pathologies and proposed mechanisms of AMD, but little about the heterocellular diversity of the choroid-RPE-retina complex or of the fates of neurons in the inner retina. The same was true in RP and models of RP where the discovery of retinal remodeling in over 30 separate models of RP was uniquely attributable to studies that employed CMP as a means to interrogate complex retinal tissues (Jones et al., 2003, 2005, 2006, 2011, 2012; Marc and Jones, 2003; Marc et al., 2003, 2005, 2007, 2008; Jones and Marc, 2005). Because inflammation plays a clear role in the etiology of early AMD (Hageman et al., 2005), our perspective was that CMP had the potential to identify early, pre-cell death signals that are uniquely associated with disease progression as CMP has been used to identify and track heterogeneous cell stress responses in inflammatory lung disease, and identify cell status in tissues prior to the time that clear differences in gene or protein expression are observed (Jean et al., 2003) and has the promise of providing insight into the dynamics of protein transporters (Pow and Robinson, 1994; Sarthy et al., 2005) and enzymatic activity (Pow and Crook, 1996; Marc et al., 1998a,b).

### RPE

Normal macular RPE are taller and narrower than those in periphery (Streeten, 1969) allowing denser RPE cellspacking, which appears necessary to maintain a low cone to RPE cell ratio (Gao and Hollyfield, 1992). With increasing age some have described the RPE as undergoing thinning and loss of cells, but there is some disagreement as to the regional distributions of these changes. Though other work has demonstrated rounding and stacking of RPE, particularly at the borders of geographic atrophy (Sarks et al., 1988; Vogt et al., 2011; Rudolf et al., 2013; Bird et al., 2014). One study found that while RPE cells were lost in large numbers in the periphery of the human retina, macular regions failed to show any significant change (Gao and Hollyfield, 1992) while another study found that the overall RPE to photoreceptor ratio dropped with age throughout the retina (Dorey et al., 1989). Notably, cell-to-cell heterogeneity is observed in nearly every measured parameter in aging human RPE, and the degree of heterogeneity appears to increase with age (Burke and Hjelmeland, 2005).

While there have been some reports that RPE cells can divide (Al-Hussaini et al., 2008; Kokkinopoulos et al., 2011), the evidence is sparse and it is clear that any cell division that might be present does not occur at a fast enough rate to compensate for disease processes that kill RPE cells in AMD. One hypothesis for progression of dry forms of AMD is that as RPE cells die, the photoreceptors internal to RPE become stressed and subsequently die. Another hypothesis is that cell death within the RPE might lead to redistribution of the remaining RPE cells and that the natural conclusion would be that the numbers of RPE cells remaining would at some point fail to have enough numbers to sufficiently redistribute, leading to breaks in the RPE such as those characteristic of more severe forms AMD. Extending that line of thought, it is tempting to hypothesize that RPE cells die faster in the central retina, causing the AMD phenotypes to be reached sooner in the central retina than in the periphery, but no data currently exists regarding the size, number, and position of RPE cells in aging human retinas that can be conclusively tied to retinal pathologies (Boulton and Dayhaw-Barker, 2001; Bonilha, 2008). That said, other studies have found no loss of RPE cells with age, even with lipofuscin-attributable autofluorescence (Ach et al., 2014). While CMP reveals more bricking in the RPE of central retina than in the periphery (Figure 2) suggesting that whatever mechanism is at work, central RPE cells have profiles that suggest greater metabolic stress in the center of the retina vs. the periphery and this correlates with pathological changes observed in aging and AMD in the retinas of human patients with AMD (Bonilha, 2008). Regardless of whether the RPE cells persist in normal aging or undergo cell death pathways in disease processes, aberrant signatures reveal altered metabolic status, possibly indicating cell stress pathways that may lead to neural retinal stress and subsequent pathologies.

The increases in glutathione in central retina of AMD subjects examined perhaps is reflective of the idea that the central retina is subject to higher levels of oxidative stress than in the periphery (Provis et al., 2005). Glutathione concentrations are also heterogeneous in the RPE of AMD patients, likely due to the decoupling patterns of RPE cells. If RPE cells are becoming uncoupled in disease, individual metabolic envelopes might be allowed to find new metastable states, or the new metabolic signatures may reflect RPE cells. Metabolite fluctuations imply decoupling, either by gap junctional conductance modulation or alternatively, downregulation of connexin expression profiles. Notably, bricking of RPE cells has also been observed in animal models of other retinal degenerative diseases (Marc et al., 2008).

Increased RPE glutathione may be an important marker of retinal stress (Marc et al., 2008). Specifically, the increase observed in glutathione in RPE cells occurs in focal regions immediately associated with both wet and dry lesions as well as in focal lesions from other disorders reflected in the Müller glia (Marc et al., 2008). With respect to the RPE, it should be noted that the human fovea lacks the retinal vascular arcades present elsewhere in the neural retina (Snodderly et al., 1992), meaning that any condition that distances the photoreceptors and neural retina from the RPE or the underlying choroid, distances the neural retina from from its only blood supply (Provis et al., 2005). Additionally, cultured RPE cells can bidirectionally transport glutathione (Lu et al., 1995), exhibit polarized transport mechanisms (Li et al., 2011) and possess a Na+-dependent transport mechanism on the apical surface of non-transformed human RPE cultures (Kannan et al., 2001), but it remains unknown what role this plays in the retina *in vivo* (Davidson et al., 1994; Lu et al., 1995; Kannan et al., 2001). Though hypoxic insult has been linked to oxidative stress in a variety of cell types, it is possible that the increase in glutathione in these cells is a response to the increased oxidative burden experienced during lesion-induced hypoxia (Sternberg et al., 1993; Schulz et al., 2000).

Glutathione is important in preventing lipid oxidation and can also detoxify reactive aldehydes, both of which are critical oxidative defenses in the RPE and photoreceptors. However, despite the prominence of oxidative damage in AMD research and the importance of glutathione in cellular oxidative homeostasis, very little is known about the role of glutathione in the retina proper of AMD retinas (Winkler et al., 1999). A number of studies have examined serum levels of glutathione in AMD patients, (Brantley et al., 2012a,b), but resolving glutathione to specific cells or cell classes has not been done prior to this study. While cultured RPE cells are protected from oxidative insults by exogenous glutathione administration and by inducers of intracellular glutathione synthesis, it is not known whether glutathione levels are changed in the RPE in AMD (Sternberg et al., 1993; Winkler et al., 1999). This study demonstrates that glutathione levels do change, but on a cell to cell basis within the RPE with wide variance of metabolic concentrations at earlier stages of AMD in RPE that suggest cell stress and possible cell death, perhaps representing cells attempting to normalize oxidative demands before collapsing late in the disease process.

Analysis of far more samples and correlation with precise diagnosis and ideally, genetic screening of genes strongly associated with AMD, e.g., CFH (Boon et al., 2009), ARMS2 (Fritsche et al., 2008; Friedrich et al., 2011) and HTRA1 (Dewan et al., 2006) as well as seven new loci associated with AMD (Fritsche et al., 2013) would be required to definitively identify whether tiling in the RPE is a normal finding of aging, or if it is associated with AMD. It could be that the tiling in the RPE indicates a cell stress mechanism that eventually results in photoreceptor cell death, leading to AMD. It could also be that the RPE stress itself may be subsequent to other disease progression mechanisms such as the “oil spill model” (Curcio et al., 2011) where cholesterol-rich lipoproteins needed by photoreceptors are taken up by the RPE via plasma, then exported to Bruch’s membrane which, over time accumulates and becomes cross-linked, effectively fixing them in place, building up into drusen that itself becomes cytotoxic and proinflammatory. It could be that we are seeing the metabolic results of cell stress brought on by these “oil-spill” deposits along Bruch membrane.

### Photoreceptors

The observation of large deposits, rich in taurine in the very distal outer segments of cone photoreceptors and material underneath the RPE (Figures 2, 6), likely represent photoreceptor material not yet phagocytosed by the RPE. Theses structures are bound by a cone opsin immunoreactive membrane containing very high concentrations of taurine and other small molecules. At the same time, rod photoreceptor opsins in these regions do not appear delocalized and are not building up material underneath the RPE. It is true that in some regions there is rod opsin delocalization, but in those regions, cones appear intact and largely healthy. Therefore, the observation of delocalized rod opsin, we believe is an early signal and cone opsin delocalization is a later signal based upon the correlations of observations with other pathologies. It should also be noted that both rod and cone opsin delocalization in other retinal diseases have been documented for over a decade (Milam et al., 2002). Rhodopsin delocalization has been observed in other models of retinal degeneration, but rod photoreceptor debris does not build up in the sub-retinal space of AMD patients as it does in RP disorders that involve RPE processing (Jones et al., 2003, 2011, 2012; Marc and Jones, 2003; Jones and Marc, 2005).

Fundamentally, the importance of this observation is that it suggests RPE cells are becoming differentially unable to process cone outer segments while rod outer segments seem to be processed normally by RPE in these regions. Given that the opsin processing pathways are identical, there must be other aspects to the phagocytosis of rod vs. cone pathways that are being compromised in these retinas. Are cone turnover and processing pathways compromised while rod opsin pathways are intact? We are unable to say for sure based upon these observations alone, but it does raise the spectre of cell specific defects when RPE cells become compromised in AMD.

While taurine is known to be especially critical for the maintenance of normal cellular volume in excitable cells such as neurons and myocytes, and is stored in very high concentration there (Huxtable, 1992; Militante and Lombardini, 2004), these observed concentrations are much higher than normal. Taurine has been demonstrated to assist in cellular defenses to oxidative stress in a variety of ways. Increases in cellular taurine and glutathione are thought to help cells cope with increased oxidative stress and are considered markers for oxidative damage (Huxtable, 1992).

Photoreceptor taurine concentrations are among the highest in the body, and the delivery and maintenance of taurine in the retina appears to be prioritized over most other areas of the body. Animals deprived of dietary taurine deplete other tissues before retinal levels are allowed to drop, and the retina is the first tissue to be re-supplied following restoration of dietary availability. Furthermore, taurine deficiency causes rapid retinal degeneration, the severity of which is proportional to the degree of deficiency (Hayes et al., 1975; Schmidt et al., 1976). However, it is not known precisely why photoreceptors are so uniquely dependent on taurine. Some studies have found that taurine supplementation protects photoreceptors from light damage and other insults thought to be oxidative in nature, so the oxidative protection roles of taurine may also be of great importance to photoreceptors (Boldyrev et al., 1999; Keys and Zimmerman, 1999). Taurine is also known to participate in regulation of RPE phagocytosis, a process critical for normal photoreceptor function (Ogino et al., 1983). Perhaps what we are seeing is an inability to process taurine in cone photoreceptors by the RPE, or alternatively there may be another mechanism operating that is simply preventing the cone photoreceptors from undergoing phagocytosis by the RPE and taurine is building up in debris underneath the RPE.

### Glutamine Synthetase

Finally, our data conflict with previous reports on GS expression being preserved in retinal disease (Strettoi et al., 2002; Roesch et al., 2012) irrespective of whether the retinal degeneration is brought on by retinal detachment (Lewis et al., 1994), RP based mechanisms or AMD based mechanisms observed in this study. We find effectively no GS in late stage AMD, raising questions of impact on overall retinal metabolic fluxes and homeostasis. Though GS is located in Müller cell populations in normal tissues (Riepe and Norenburg, 1977), and assists the glutamate/glutamine cycle through conversion of extracellularly derived glutamate into glutamine (Riepe and Norenburg, 1977), its absence implies that the retinal microenvironment around Müller cells may no longer support cone function (Bringmann et al., 2006). Indeed, some studies that have ablated Müller glia have observed implications for photoreceptor survival (Shen et al., 2012). Though that study selectively ablated Müller cells completely, one might imagine a more selective impact on GS might lead to similar results.

## Summary

This manuscript adds to the literature supporting alterations of the RPE including RPE cell thinning underneath drusen and alterations observed in aging/diseased human retinas and ties these changes to the presence of other subsequent retinal changes and most notably, retinal remodeling. These changes are by definition, pathology and ultimately result in a progressive, irreversible neural degeneration reflected by loss of retinal neurons and glia in AMD. The pathologies begin with the earliest indications of cell stress identified early through metabolic instability in the RPE and in photoreceptors as well as Müller glia. Additionally, cone opsin processing by the RPE appears to be differentially impacted through an inability to process cone opsin bound materials that build up underneath the RPE. Neural retinal remodeling/plasticity in AMD is observed through aberrant sprouting of amacrine cell processes and likely other processes as well. This remodeling occurs early, particularly underneath drusen and contrary to widely held belief, is a likely contributor to visual loss even before photoreceptor cell loss when both rod and cone photoreceptors are still present.

Before we can hope for long-term positive outcomes from retinal vision rescues of all kinds, the vision science community needs to address basic mechanisms for plasticity in that any rescue of vision whether via RPE replacement, photoreceptor replacement via biological or bionic methods or gene therapy will be compromised in some form by ongoing retinal plasticity. The mechanisms at play in AMD will be particularly difficult given the number of potential targets involved, but there may be common pathways that lead to either cell survival or restraining of plasticity by both neurons and glia.

## Author Contributions

BWJ designed approach, generated primary manuscript, analyzed data and generated figures. REM contributed to experimental design, primary manuscript and data interpretation. RLP, WDF, JT collected data, participated in CMP and OCT imaging. CBW collected TEM imaging. RLP assisted with manuscript preparation and figure generation.

## Conflict of Interest Statement

The authors declare that the research was conducted in the absence of any commercial or financial relationships that could be construed as a potential conflict of interest. REM is a principal of Signature Immunologics.

## Abbreviations

AMD: age-related macular degeneration
RP: retinitis pigmentosa
RPE: retinal pigment epithelium
D: aspartate
R: arginine
E: glutamate
G: glycine
J: glutathione
Q: glutamine
τ: taurine
1D4: rod opsin
rg-opsin: cone opsin
GS: glutamine synthetase
GABA: γ-aminobutyric acid
CRALBP: Retinaldehyde binding protein 1
IgG: immunoglobulin G
EM: electron microscopy
rgb: red green blue
ONL: outer nuclear layer
ISODATA: Iterative Self-Organizing Data Analysis Technique
CMP: Computational Molecular Phenotyping
μM: micrometer.
